# Acute retinal necrosis associated with dimethyl fumarate

**DOI:** 10.1177/13524585251326475

**Published:** 2025-05-04

**Authors:** Christopher Paisey, Karen Curtin, Simon J Epps, Claire M Rice

**Affiliations:** Southmead Hospital, North Bristol NHS Trust, Bristol, UK; Bristol Eye Hospital, University Hospitals of Bristol and Weston NHS Foundation Trust, Bristol, UK; Bristol Eye Hospital, University Hospitals of Bristol and Weston NHS Foundation Trust, Bristol, UK; Southmead Hospital, North Bristol NHS Trust, Bristol, UK; Translational Health Sciences, University of Bristol, Bristol, UK

**Keywords:** Dimethyl fumarate, acute retinal necrosis, varicella zoster virus, relapsing remitting multiple sclerosis, optic neuritis

## Abstract

**Background:**

The full range of side effects of disease-modifying treatment for multiple sclerosis (MS) remains to be determined. To date, four serious varicella zoster virus (VZV) infections have been reported in association with dimethyl fumarate (DMF).

**Report:**

A 44-year-old man on DMF for relapsing remitting MS presented with monocular pain and visual impairment. Ophthalmological examination revealed panuveitis with arteritis, retinitis and vitritis. Vitreous PCR was positive for VZV confirming the clinical impression of acute retinal necrosis (ARN).

**Discussion:**

ARN due to VZV may mimic optic neuritis in people on disease-modifying therapy for MS.

## Background

People with multiple sclerosis (MS) may present with painful loss of vision due to optic neuritis. However, the presentation warrants careful assessment and consideration of the differential diagnoses. When there is concomitant immunomodulation due to disease-modifying therapy, the possibility of an underlying infective aetiology should be given particularly careful consideration.

We present the case of a 44-year-old man with acute retinal necrosis (ARN) attributable to varicella zoster virus (VZV) infection in the context of disease-modifying therapy with dimethyl fumarate (DMF).

## Case report

**Figure 1.**
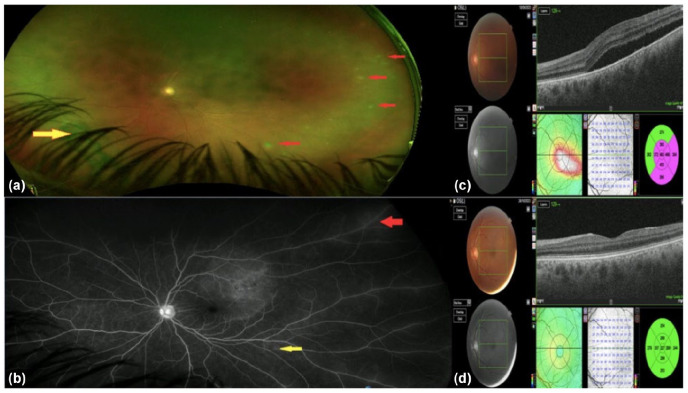
Acute retinal necrosis. Wide–field Optos® photo of the left eye (a) showing multifocal areas of retinitis (red arrows) and an inferonasal area of pre-retinal haemorrhage (yellow arrow). Wide-field fundus fluorescein angiogram (b) showing multiple areas of vasculitis. Red arrow indicating venulitis and yellow arrow showing arteritis. Optical coherence tomography (OCT) at presentation (c) showing large volume sub-retinal fluid in the left eye, which has resolved completely by the sixth week of treatment (d).

A 44-year-old man presented to the emergency ophthalmology service with left conjunctival injection, mild eye pain and impaired vision. He was a non-smoker with a history of cluster headache. For the preceding 6 years, he had been on DMF as disease-modifying therapy for MS. The Expanded Disability Status Score was 4.5, and a recently updated magnetic resonance (MR) head scan had not shown any new inflammatory changes. The initial ophthalmological review noted the presence of sub-retinal fluid (SRF) at the left macula of uncertain aetiology. He represented 1 week later with worsening symptoms of photophobia, ocular discomfort and deteriorating vision. Visual acuity was 47 letters (Early Treatment of Diabetic Retinopathy Study; 0.76 LogMAR; approximately 6/36 Snellen) in the left eye and 85 letters (0 LogMAR; 6/6 Snellen) in the right. He had left-sided panuveitis with retinal arteritis and venulitis, and multifocal areas of retinitis consistent with ARN (Figure 1). Although the lymphocyte count had been intermittently low on DMF (lowest recorded 0.7 × 10^9^/L), blood results including lymphocyte count were normal at the time of presentation. Screening for HIV, syphilis and tuberculosis was negative. Vitreous PCR was positive for VZV. Treatment was commenced with cyclopentolate and prednisolone acetate eye drops, 60 mg oral prednisolone (started 48 hours after antiviral treatment) and valaciclovir, as well as intravitreal foscarnet. DMF was discontinued. By the sixth week of treatment, left-sided acuity was 77 letters (0.16 LogMAR; approximately 6/9 Snellen), sub-retinal fluid and intraocular inflammation had fully resolved, and an area of inferonasal retinal haemorrhage was receding (Figure 1).

## Discussion

ARN is a rare (incidence 1/1.6–2 million/year), sight-threatening panuveitis associated with herpesvirus infection, most commonly VZV.^1–3^ Prompt treatment minimises intraocular complications and risk to the contralateral eye. Increasing age and immunosuppression are risk factors for ARN, attributed to a decline in VZV-specific T-cell-mediated immunity.^4^

DMF is known to deplete T-cells, particularly CD8+ cells,^5^ and four cases of severe herpes zoster have been reported; one had 60% reduction in peripheral blood lymphocytes and the others had reduced CD8+ T-cell counts.^6–8^ Recently, increased CD4+:CD8+ ratios have been shown to be associated with herpes zoster in people treated with DMF.^9^ To the best of our knowledge, this is the first reported case of ARN in a young person on DMF. His absolute lymphocyte count was normal, but T-cell subsets were not documented acutely.

The diagnosis of ARN was made based on the clinical trial of arteritis, retinits and vitritis. Although epiretinal membrane formation and cystoid macular oedema are well-recognised complications, the presence of SRF is atypical. Alaghband et al.^10^ described a 40-year-old Caucasian with VZV-related ARN and serous macular detachment. They hypothesised that SRF accumulated due to inflammation of the retinal pigment epithelium (RPE) and breakdown of the RPE pump.

## Conclusion

Given the risk to vision and requirement for prompt antiviral therapy in ARN, our case highlights the importance of considering causes other than optic neuritis (ON) for reduced visual acuity in people with MS. The absence of a relative afferent pupillary defect or colour vision deficit should prompt careful ophthalmological assessment, and the features of retinal vasculitis, retinitis and vitritis should lead to consideration of an infective aetiology. While an episode of ON typically features pain on eye movement, ocular pain associated with anterior uveitis is usually accompanied by conjunctival injection and photophobia, and these features should highlight the likelihood of an alternative diagnosis.
